# Growth and Fabrication of High External Quantum Efficiency AlGaN-Based Deep Ultraviolet Light-Emitting Diode Grown on Pattern Si Substrate

**DOI:** 10.1038/s41598-017-11757-1

**Published:** 2017-09-22

**Authors:** Binh Tinh Tran, Hideki Hirayama

**Affiliations:** 1 RIKEN Center for Advanced Photonics, 2-1 Hirosawa, Wako, Saitama, 351-0198 Japan; 2 Department of Electrical Engineering and Computer Science, University of Michigan, 1301 Beal Avenue, Ann Arbor, MI 48109-2122 United States; 3 Quantum Optodevice Laboratory, RIKEN, 2-1 Hirosawa, Wako, Saitama, 351-0198 Japan

## Abstract

Growing III-V semiconductor materials on Si substrates for opto-electronic applications is challenging because their high lattice mismatch and different thermal expansion coefficients cause the epitaxial layers to have low quality. Here we report the growth of a high-quality AlN template on a micro-circle-patterned Si substrate by using NH_3_ pulsed-flow multilayer AlN growth and epitaxial lateral overgrowth techniques. Then, we fabricated and characterized a deep-ultraviolet light-emitting diode (UV-LED) device using this AlN/patterned Si. By using standard lithography and inductively coupled plasma etching, the Si substrate was prepared with very high pattern density and was made deep enough to grow a thick AlN template with high crystal quality and very few threading dislocations, allowing for further re-growth of the deep UV-LED device. And by combining a transparent p-AlGaN contact layer, an electron blocking layer and using this high quality AlN template: a deep UV-LED device fabricated and showed a strong single sharp electroluminescence (EL) peak at 325 nm and achieved an external quantum efficiency (EQE) of about 0.03%, for a deep UV-LED grown on Si substrate.

## Introduction

AlGaN-based deep UV-LEDs with emission wavelengths from 200 to 360 nm have many important applications in environmental, industrial, medical, and life sciences for water and air disinfection, sensing, medical curing, printing, counterfeit detection, and other areas^1–4^. To date, commercial deep UV-LEDs for the living environment, which have been developed on only expensive substrates such as sapphire, display very high EQE values (depending on the emission wavelength)^1,5^ due to improvements in the crystal quality, electron injection current, and light extraction efficiency as well as optimization of structures^1,6–8^. However, developing deep UV-LEDs based on Si substrates is challenging: the growth of the AlN template, which is necessary to include as a layer on the Si substrate, generally suffers from cracking due to the different thermal expansion coefficients of Si and AlN. Moreover, AlN/Si contains a high threading dislocation density due to the large lattice mismatch between the AlN and Si components^9–11^. Although we have been able to grow a crack-free AlN template on a flat Si substrate layer with thickness of less than 1 µm, it has not been possible to obtain a high crystal quality and low threading dislocation density simultaneously for such a thin AlN template. Therefore, the best choice has been to use a patterned Si substrate since a thick AlN template with a high crystal quality can be grown on this type of substrate, and recently a few groups have obtained very good results in this regard, and such a process could be used to further develop deep UV-LEDs^12–14^. For these reasons, and because Si substrates are inexpensive, show good thermal conductivity and are available in very large sizes and possibility of integrating LEDs on Si devices;^15,16^ the development of deep UV-LEDs based on Si substrates shows considerable promise.

The greatest remaining challenge for the development of deep UV-LEDs on Si substrates, however, may be its low light extraction efficiency^17^, which is due to several factors. One factor is the unique optical polarization properties of the spontaneous emission from the active layer, i.e., AlGaN multi-quantum wells (MQWs) emit light with transverse-magnetic polarization along the direction normal to the surface, leading to the low efficiency. Note that the emission of TM-polarized light from the AlGaN MQWs is significantly dependent on the amount of Al in the MQWs^17–19^. The second reason is the different reflective indexes of the p-type layer to the p-metal electrode on the front side and the LEDs to the air on the back side emission. A third reason may be that the p-GaN typically used for the contact layer strongly absorbs the light emitted from the AlGaN MQWs, resulting in low light extraction from the surface^18,20,21^. For these reasons, there have been very few reports about the development of deep UV-LEDs on Si substrates with high quantum efficiency and wavelengths shorter than 350 nm, which requires high Al content.

In this paper, we report on the growth, fabrication, and characterization of a deep UV-LED on patterned Si substrate with an emission wavelength belongs to UVA. The metal–organic chemical vapor deposition reactor (MOCVD) grows an AlN template on a micro-circle-patterned Si substrate (mPSiS) by using a combination of NH_3_ pulsed-flow multilayer AlN growth and epitaxial lateral overgrowth techniques. The details of pattern fabrication and growth methods can be found somewhere^14,22^. The AlN template was then placed in another MOCVD reactor to regrow a deep UV-LED structure and fabricate a device with an emission wavelength of 325 nm and high EQE.

## Results and Discussion

**Figure 1:**
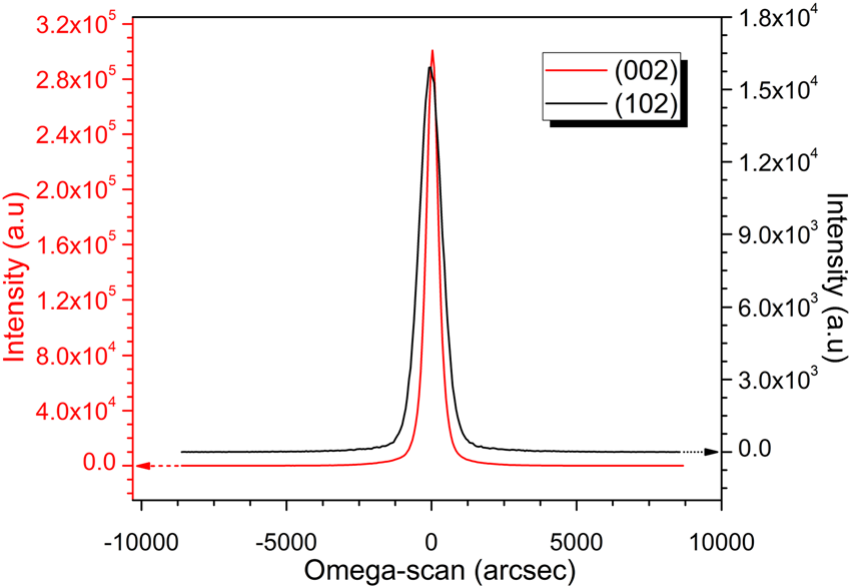
XRD rocking curve of the AlN template grown on a micro-circle-patterned Si substrate with FWHM values of 620 arcsec (002) and 1141 arcsec (102) reflection planes. As we mentioned in the introduction; Hence, the sample used to carry out the fabrication and measurement for this paper is also the sample that was used in the Ref.^14^.

Figure 1 shows XRD FWHM rocking curves of an 8-µm-thick AlN template grown on a micro-circle-patterned Si substrate at 1380 °C. The FWHM values of the XRD peaks corresponding to the (002) and (102) reflection planes were measured to be 620 arcsec and 1141 arcsec, respectively. These values are, to our knowledge, the lowest reported (002) and (102) FWHM values for an AlN grown on a Si substrate. These low values may have been due to the high pattern density with a small edge-to-edge distance of the patterns^22^. Thus, the quality of this AlN crystal was greatly improved, by about 25%, compared with that in our previously report^14^ and much lower than the crystal quality in other current reports^10,12,23,24^.

**Figure 2:**
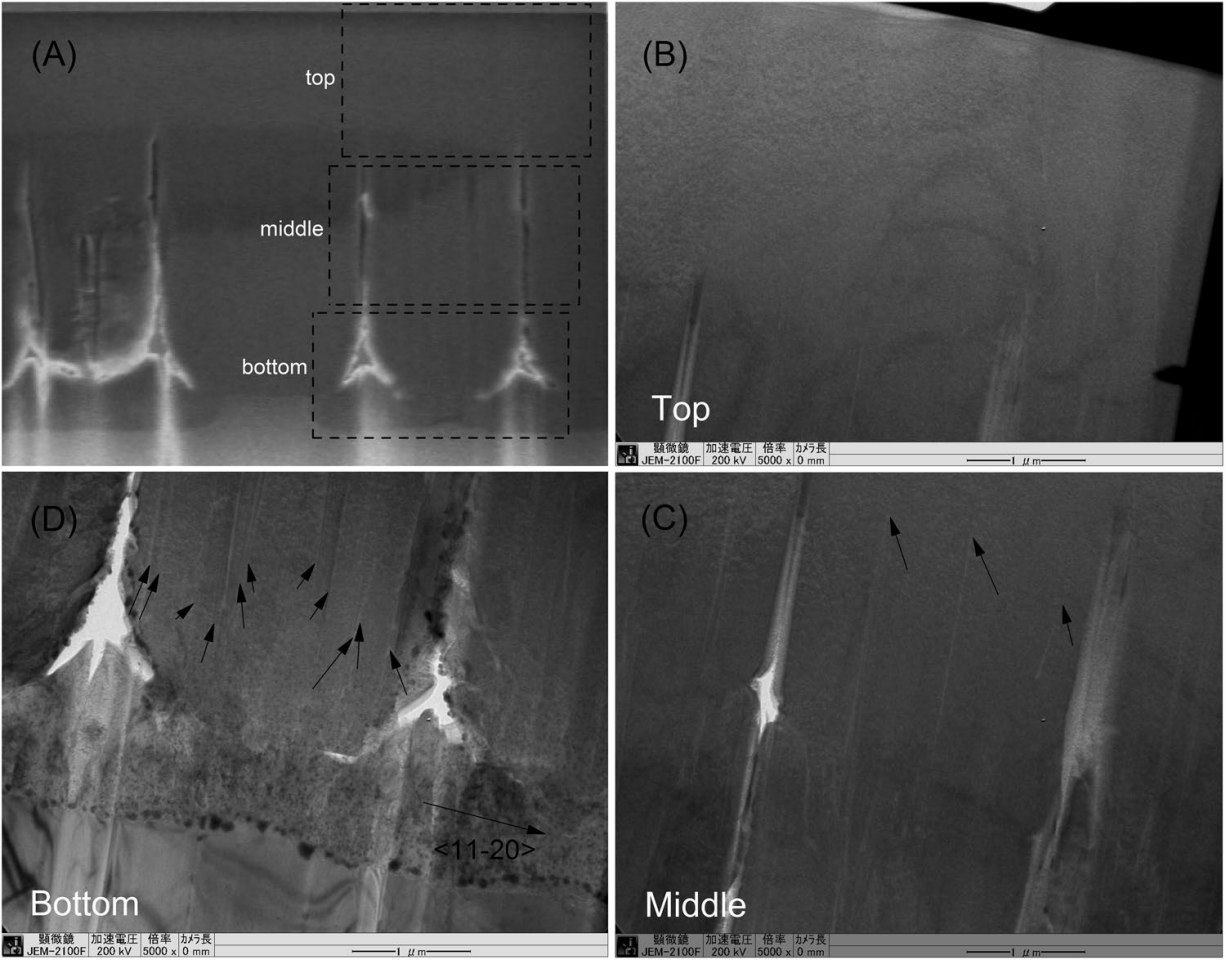
(**A**) Cross-sectional SEM image of the AlN template taken after using focused ion beam processing. A thick completely coalesced on the top of the AlN template can be seen (where the fifth AlN layer started to grow). (**B-D**) Corresponding TEM images revealed many dislocations near the bottom of the AlN template, but almost none on the top. They are very similar to the results of Ref.^14^ since we took them at a nearby position.

To determine the coalescence thickness as well as the threading dislocation density of the AlN template grown on the mPSiS, we first used a focused ion beam technique to cut the sample as shown in Fig. 2 A According to the cross-sectional SEM image, coalescence began at the end of the third AlN layer, and the fourth and fifth (i.e., last) layers were completely coalesced without any voids inside. These completely coalesced layers were also observed to be very thick (i.e., more than 3.0 µm). This result indicates that the XRD FWHM of this AlN template was an excellent achievement. The effect of AlN quality on the UV-LEDs performance can be found somewhere^14,22^.

Cross-sectional TEM images of the AlN/patterned Si substrate were taken with g =  < 11–20 > and showed the dislocation densities to be different in different regions of the sample. Many dislocations were observed in the bottom of the template (Fig. 2 D), although fewer in its middle (Fig. 2 C) where some dislocations terminated and most of them could not reach the surface. Therefore, very few dislocations were seen in fifth (top) layer of the AlN template (Fig. 2 B). As estimated results, the dislocation densities were estimated to be about 5 × 10^7^ cm^−2^ (screw) and 7.5 × 10^7^ cm^−2^ (edge) in the top region, and on around the order of 10^9^ cm^−2^ in the middle and bottom regions.

**Figure 3:**
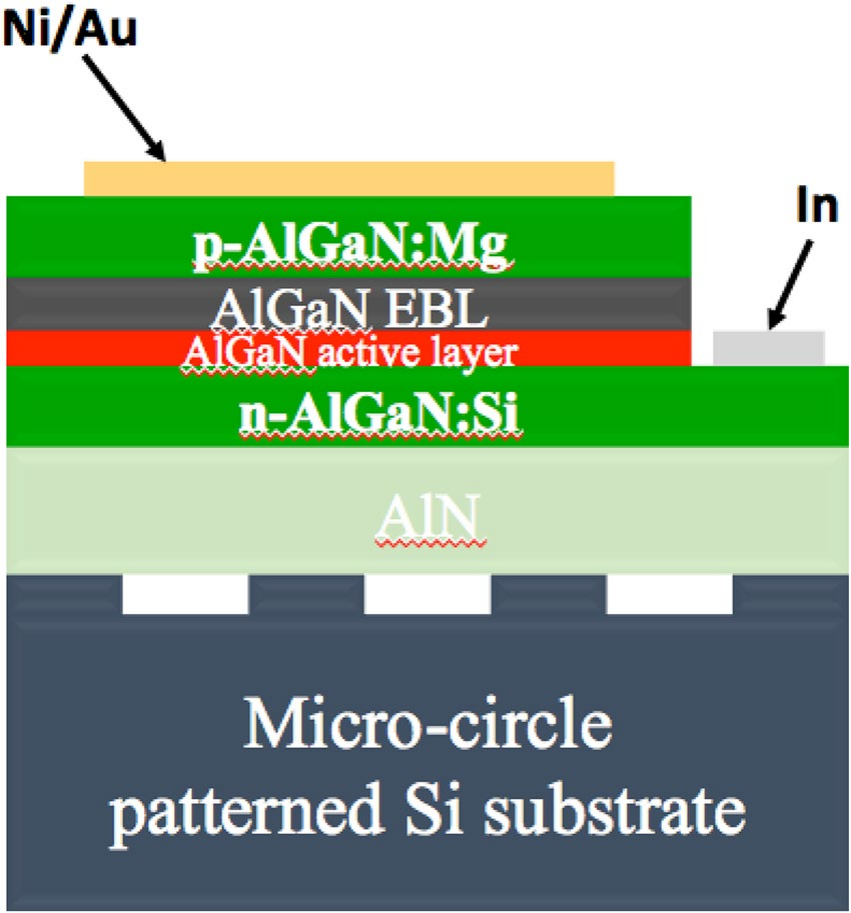
Schematic of the deep UV-LED structure grown on the micro-circle-patterned Si substrate with a transparent p-AlGaN layer and an electron-blocking layer.

**Figure 4:**
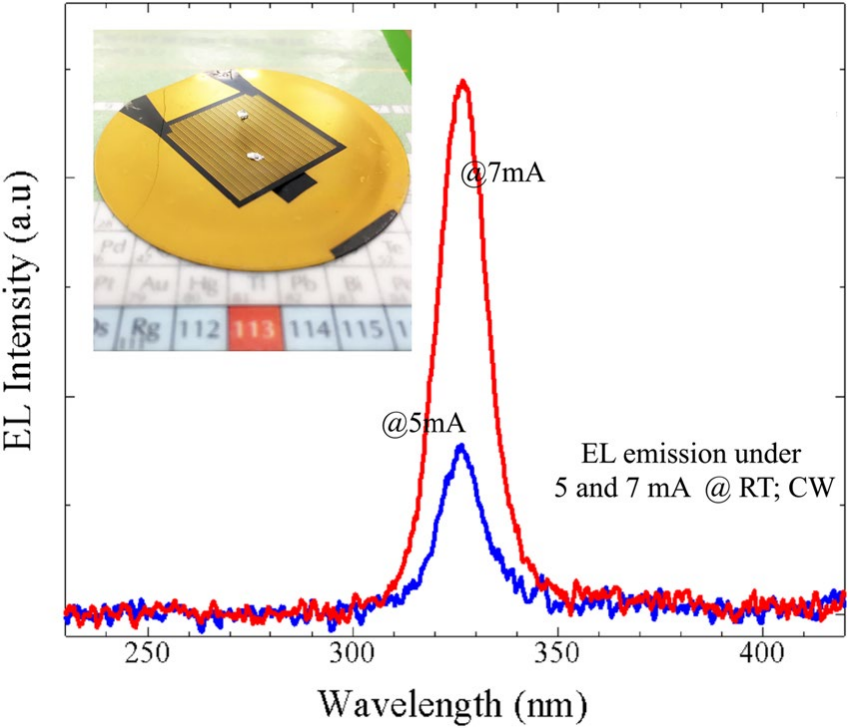
The electroluminescence spectrum was measured at room temperature under CW operation and various current injections. A single sharp peak without any shoulders was observed at a wavelength of 325 nm. The inset figure is the deep UV-LED device which was grown and fabricated on Si substrate and used to measure and present the data in this work.

The AlN/patterned-Si substrate was then used to grow and fabricated into a deep UV-LED device, with the schematic of this device shown in Fig. 3. First, we loaded the substrate into another MOCVD reactor to grow the deep UV-LED structure with an approximate 1.3 µm n-Al_0.6_Ga_0.4_N layer by doping Si, and then a three-undoped Al_0.2_Ga_0.8_N/Al_0.7_Ga_0.3_N MQWs/barriers layer was continuously grown with about 2 nm and 6 nm for each well and barrier layer, respectively. A 25 nm p-Al_0.95_Ga_0.05_N layer doped with Mg was used as an electron-blocking layer (EBL), and finally, an approximately 100 nm transparent p-Al_0.7_Ga_0.3_N layer was grown on the top for the contact layer with the p-type doping concentration to be expected at about 10^16^/cm^3^. All these layers were grown using a low-pressure MOCVD reactor with detailed growth conditions are listed in Table 1. The p-AlGaN contact layer was grown at 1150 °C, and the other layers at 1200 °C. The growth pressure is kept in constant for all layers at 76 Torr. After the growth, the sample was heated at 850 °C in a N_2_ atmosphere for 50 min. Ni/Au metal and In balls were then used as p- and n-contact electrodes, respectively. The thickness of the Ni and Au components of the p-electrode were about 15 nm and 25 nm, respectively, and the final device is shown in the inset of Fig. 4. The transparent p-AlGaN layer was one of keys contributor to improving the light extraction efficiency of the deep UV-LED. The transmittance of the p-AlGaN with about 70% Al composition has been carried out^6^ and found that transmittance ratio exceeded 95% for the wavelength range from 250 nm to 400 nm.

**Table 1:** Growth conditions of a deep UV-LED on patterned Si substrate.

Layer	n-AlGaN	Wells/Barriers	EBL	p-AlGaN
Temp (°C)	1200	1200	1200	1150
Time (min)	116	21 sec/34 sec × 3	37 sec	10
Press (Torr)	76	76/76	76	76
TMAl (sccm)	20	6/20	16	12
TMGa (sccm)	4	5/5.4	3	4
N_2_ (liter)	3.5	3.5/3.5	3.5	3.5
H_2_ (liter)	5	5/5	5	5
NH_3_ (liter)	2	2/2	2	4
Mg-dopant (sccm)	0	0.0/0.02	30	70
Si-dopant (sccm)	0.1	0	0	0

**Figure 5:**
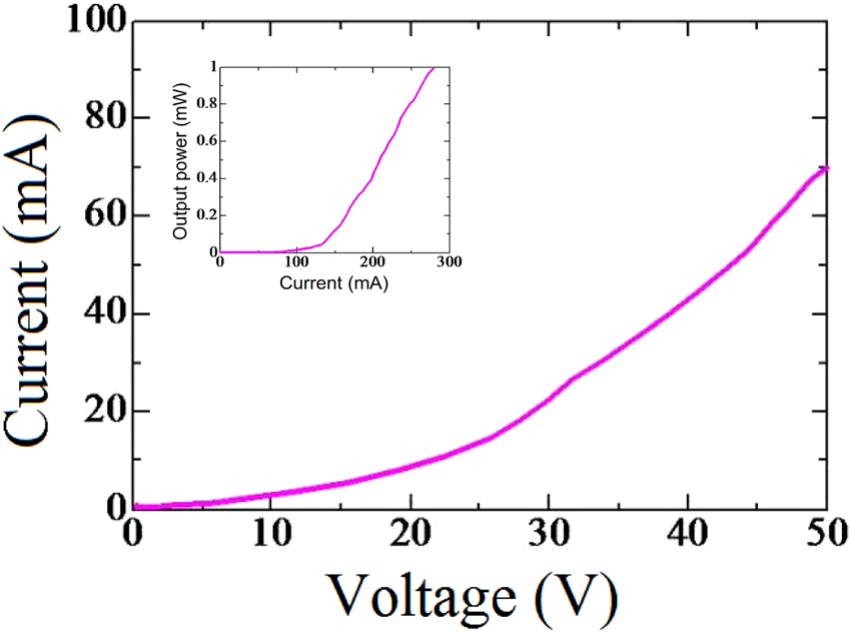
I-V characteristics and output power (inset) of the front side of the deep UV-LED device measured at room temperature.

An electroluminescence spectrum was acquired at room temperature under continuous-wave (CW) operation with a Si photodetector to measure the output power. Before acquiring the spectrum, the system was calibrated by measuring the luminous flux from an LED source by using an integrating sphere system. Figure 4 shows the electroluminescence spectrum of the deep UV-LED grown on the AlN/patterned Si substrate at 1200 °C for the bare wafer condition (see the inset of Fig. 4) with a single sharp peak at 325 nm was clearly obtained under different currents. The EL emission during the measurement is shown in the supplementary information. We provided that supplementary information because the EL spectrum of a deep UV-LED device identical to our current device but using p-GaN instead of p-AlGaN for the contact layer did not show this peak. (Perhaps the p-GaN layer, because it absorbs light, yielded a light extraction efficiency too low to be observed in its EL spectrum). The output power at room temperature was also measured under CW mode and is shown in the inset of Fig. 5. The deep UV-LED wafer was placed into a calibrated system and its output power was measured by using an optical meter. A low output power was measured at an injection current of 500 mA and plotted in the inset of Fig. 5. In particular, an EQE of 0.03% was achieved, the highest value for a deep UV-LED grown on a Si substrate at the recorded wavelength of 325 nm. This EQE value is considered to be very high for deep UV-LED/Si since this value is many times higher than the latest reported value (but at different emission wavelength)^25^. It is, however, much lower than that of deep UV-LED/sapphire, because the Si substrate is a black body and hence completely absorbs incident light, resulting in the light extraction efficiency from the front emission being very low. The low output power was also due to the p-electrode being blocked off and the UV light being extracted from only the narrow edge of the p-electrode. Thus, removing the Si substrate to realize emission from the back side is expected to increase the light extraction efficiency and output power significantly^12,26^.

## Conclusions

In summary, we have produced a 325-nm-wavelength AlGaN-based deep UV-LED, which was grown on a micro-circle-patterned Si substrate for the first time, with AlN template XRD FWHM rocking curves of 620 and 1141 arcsec for the (002) and (102) reflection planes, respectively. The high crystal quality of the AlN template with its very low dislocation density and absence of cracks proved that using the NH_3_ pulsed-flow multilayer AlN growth and epitaxial lateral overgrowth techniques with multiple growth steps could improve the quality of the AlN template significantly. The successful growth and fabrication of the deep UV-LED on the micro-circle-patterned Si substrate also demonstrated the benefit of the high crystal quality of the AlN template with a very high EQE proved. The EQE value reported here, while very lower than that of deep UV-LEDs grown on sapphire substrates, nevertheless proved that it is possible to fabricate deep UV-LEDs grown on Si substrates, and this result provides a strong motivation to improve the device performance of deep UV-LEDs on low-cost Si substrates.

## Methods

The deep UV-LED device was developed using an AlGaN alloy and grown on an AlN/patterned Si substrate. MOCVD was carried out in a low-pressure reactor and trimethylaluminum, trimethylgallium, and ammonia were used as sources of Al, Ga, and N, respectively. Si and Cp_3_Mg were for n- and p-type doping. The thick AlN template (about 8 µm thick) included 5 sub-AlN layers grown at 1380 °C. The first thin AlN layer was grown as an interlayer between the upper layers and the patterned Si substrate. This layer was grown for 10 min at 200 Torr. The second layer was grown for 11 min at 76 Torr. The second, third, fourth, and fifth AlN layers were grown at the same pressure in order to obtain coalescence at the end of the third layer. The third, fourth, and fifth AlN layers were grown for 60, 8, and 60 min, respectively. The fourth layer was used to reduce the threading dislocation density of the fifth layer, and was also coalesced to serve as the main layer of the AlN template. The first, second, and fourth AlN layers were grown using the NH_3_ pulsed-flow multilayer AlN growth technique; meanwhile, the third and fifth AlN layers were grown using the epitaxial lateral overgrowth technique. To evaluate the crystal quality of the AlN template, X-ray diffraction (XRD), scanning electron microscopy (SEM), and transmission electron microscopy (TEM) were carried out to determine the XRD full width at half-maximum (FWHM), coalescence thickness, and threading dislocation density, respectively.

## Electronic supplementary material

UV-LED/Si under EL measurement

The AlGaN-based deep UV-LED grew and fabricated on a pattern Si substrate.
