# Supplementary material for: Growth and Fabrication of High External Quantum Efficiency AlGaN-Based Deep Ultraviolet Light-Emitting Diode Grown on Pattern Si Substrate
Source: Sci Rep. 2017 Sep 22;7:12176. doi: 10.1038/s41598-017-11757-1 (PMC5610239; doi:10.1038/s41598-017-11757-1)
Supplement: Supplementary file 2 — The AlGaN-based deep UV-LED grew and fabricated on a pattern Si substrate. [file 41598_2017_11757_MOESM2_ESM.doc]

**Growth and Fabrication of High External Quantum Efficiency AlGaN-Based Deep Ultraviolet Light-Emitting Diode Grown on Pattern Si Substrate**

Binh Tinh Tran^1,2^* and Hideki Hirayama^1,3^

*^1^RIKEN Center for Advanced Photonics, 2-1 Hirosawa, Wako, Saitama 351-0198, Japan.*

*^2^Department of Electrical Engineering and Computer Science, University of Michigan,*

*1301 Beal Avenue, Ann Arbor, MI 48109-2122, United States.*

*^3^Quantum Optodevice Laboratory, RIKEN, 2-1 Hirosawa, Wako, Saitama 351-0198, Japan.*

**Corresponding author:* [*tinh.tran@riken.jp*](mailto:tinh.tran@riken.jp); on the leave to: [*tinhtran@umich.edu*](mailto:tinhtran@umich.edu)

**Supplemental materials**

As mentioned in the manuscript, the AlGaN-based deep UV-LED device has been fabricated and measured under room temperature as shown in Figure 1.

Please watch the video clip (*The Video clip proves that the UV-LED/Si works well by watching the EL measurement.*) in the supplementary information, which was recorded during the EL measurement to see its operation.


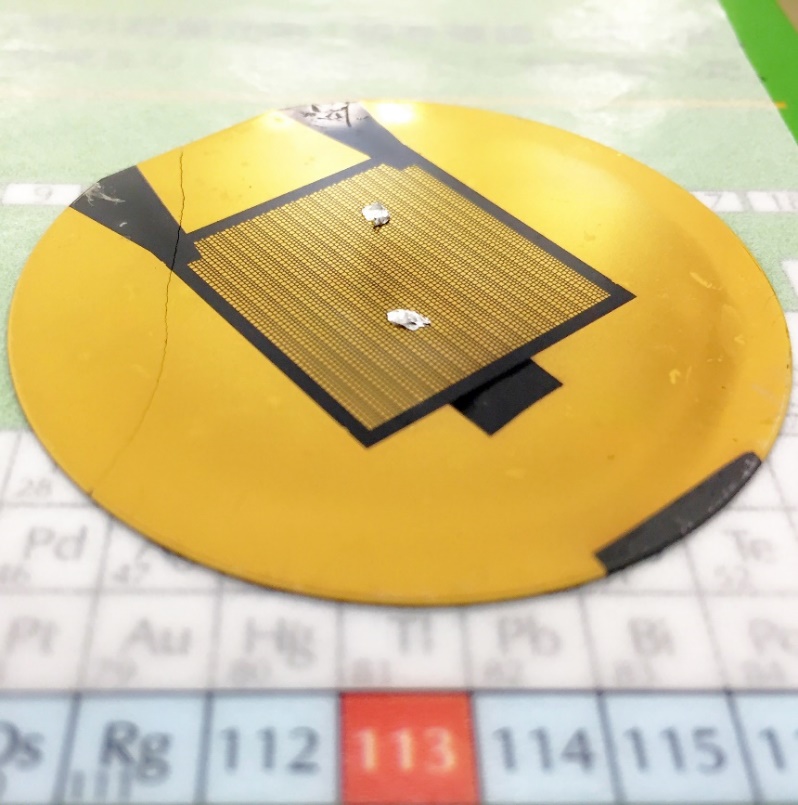


Figure 1. The AlGaN-based deep UV-LED grew and fabricated on a pattern Si substrate.
